# Unlocking Synergistic Hepatoprotection: Dapagliflozin and Silymarin Combination Therapy Modulates Nuclear Erythroid 2-Related Factor 2/Heme Oxygenase-1 Pathway in Carbon Tetrachloride-Induced Hepatotoxicity in Wistar Rats

**DOI:** 10.3390/biology13070473

**Published:** 2024-06-26

**Authors:** Shakta Mani Satyam, Laxminarayana Kurady Bairy, Abdul Rehman, Mohamed Attia, Layth Ahmed, Karam Emad, Yusuf Jaafer, Abdelrehman Bahaaeldin

**Affiliations:** 1 Faculty of Pharmacology, RAK College of Medical Sciences, RAK Medical and Health Sciences University, Ras Al Khaimah 11172, United Arab Emirates; kurady@rakmhsu.ac.ae; 2 Faculty of Pathology, RAK College of Medical Sciences, RAK Medical and Health Sciences University, Ras Al Khaimah 11172, United Arab Emirates; rehman@rakmhsu.ac.ae; 3 RAK College of Medical Sciences, RAK Medical and Health Sciences University, Ras Al Khaimah 11172, United Arab Emirates; 4 RAK College of Pharmacy, RAK Medical and Health Sciences University, Ras Al Khaimah 11172, United Arab Emirates

**Keywords:** sodium–glucose co-transporter-2 inhibitors, hepatotoxicity, liver diseases, non-alcoholic fatty liver disease, oxidative stress, repurposing, antidiabetics

## Abstract

**Simple Summary:**

This study explored the liver-protective effects of dapagliflozin and silymarin, alone and combined, against liver damage caused by carbon tetrachloride (CCl_4_) in rats. Thirty rats were divided into five groups. All groups except the normal control group were given CCl_4_ to induce liver damage. The other groups received treatments with gum acacia, silymarin, dapagliflozin, or a combination of dapagliflozin and silymarin for 14 days. The results show that both dapagliflozin and silymarin, alone and combined, significantly reduced liver damage markers in the blood compared to the group that only received CCl_4_. Additionally, these treatments lowered levels of inflammatory substances and increased antioxidant enzyme levels in the liver. The combination of dapagliflozin and silymarin was especially effective, suggesting they work well together to protect the liver. This study highlights the potential of dapagliflozin and silymarin in preventing liver damage by influencing specific protective pathways in the body.

**Abstract:**

This study was aimed to investigate the hepatoprotective potential of dapagliflozin and silymarin alone and in combination to combat carbon tetrachloride (CCl_4_)-induced hepatotoxicity and the anticipated mechanisms. Thirty female Wistar rats were randomly allocated into five different groups. All the experimental animals except the normal control (Group I) were administered CCl_4_. Additionally, Groups II, III, IV, and V were treated with gum acacia, silymarin, dapagliflozin, and a combination of dapagliflozin and silymarin, respectively, for 14 days. Dapagliflozin, silymarin alone, and in combination, significantly reduced (*p* < 0.05) serum levels of ALT, AST, AST:ALT ratio, and total bilirubin compared to CCl_4_-intoxicated control rats. There was a notable reduction (*p* < 0.05) observed in the levels of IL-1beta, IL-6, TNF-alpha, nitrites, and 4-hydroxynonenal, accompanied by an elevation in catalase, superoxide dismutase, glutathione peroxidase, nuclear erythroid 2-related factor 2 (Nrf2), and heme oxygenase-1 (HO-1) in liver homogenates of the groups treated with dapagliflozin, silymarin alone, and in combination, as compared to the CCl_4_-intoxicated control group. Dapagliflozin in combination with silymarin showed a synergistic hepatoprotective effect. Our study reveals the profound hepatoprotective potential of dapagliflozin alone and in combination with silymarin in CCl_4_-intoxicated Wistar rats by modulating the Nrf2 and HO-1 signaling pathways.

## 1. Introduction

The liver is a vital organ within the human body. It constitutes about 2% of an adult’s body weight, yet it processes approximately 25% of the total cardiac output [1]. The liver filters blood from the gastrointestinal tract, detecting and metabolizing molecu-lar signals and foreign substances. It processes and eliminates internal and external compounds, stores and regulates essential nutrients, and synthesizes vital proteins, bile acids, hormones, and regulatory molecules. The liver also supports the immune system by clearing microorganisms and toxins from the blood. It is a main target for toxic effects from foreign substances and pathogens, often damaged by metabolizing chemicals, drugs, and pollutants due to its high blood flow and metabolic activity.

Hepatotoxicity, characterized by liver damage induced by chemical substances, remains a significant global health concern. Liver disease contributes to over two million deaths annually, including those attributed to cirrhosis, viral hepatitis, and liver cancer, making up 4% of global mortality, which translates to approximately one out of every 25 deaths [2]. Among these fatalities, liver cancer alone is responsible for between 600,000 to 900,000 deaths [3]. Although liver disease currently ranks as the eleventh-leading cause of death globally, there is a possibility that the actual number of liver-related deaths is underestimated [2]. One of the studies has reported that the increase in mortality in cirrhosis is higher in comparison with other chronic conditions [4]. Liver diseases remain a critical challenge in clinical practice due to their complex pathogenesis and limited therapeutic options.

CCl₄-induced hepatotoxicity is extensively used as an experimental model to study the mechanisms of liver injury and to screen potential hepatoprotective agents [5]. A CCl₄-induced hepatotoxicity model mimics the pathophysiology of human liver diseases such as fibrosis, cirrhosis, and hepatocellular carcinoma [5,6]. The pathogenesis of CCl₄-induced hepatotoxicity involves oxidative stress, inflammation, and apoptosis, with reactive oxygen species (ROS) playing a crucial role [7]. The metabolic activation of CCl₄ by hepatic cytochrome P450 enzymes generates trichloromethyl radicals (CCl₃•), which trigger lipid peroxidation, protein oxidation, and DNA damage, leading to hepatic necrosis and fibrosis [8].

Despite significant advancements in modern medicine, liver diseases continue to be a significant public health issue, underscoring the need for new, side-effect-free medications. A significant focus has been placed on investigating therapeutic agents that can mitigate liver damage through antioxidant mechanisms [9]. Some of the studies have reported that plant extracts like silymarin, derived from the seeds of Silybum marianum, commonly known as milk thistle, are used to treat liver diseases [10,11,12,13,14]. Research has highlighted its strong hepatoprotective antioxidant properties, which are achieved by inhibiting lipid peroxidation [15,16].

The strategy of repurposing existing drugs for new therapeutic purposes is becoming more prominent in drug discovery and development due to its potential to expedite treatment availability for various medical conditions. Repurposing existing drugs offers numerous advantages, including cost savings, reduced development time, and improved patient outcomes [17].

SGLT2 inhibitors, initially developed to treat type 2 diabetes mellitus (T2DM), have garnered attention due to their multifaceted effects beyond glycemic control. Dapagliflozin, a selective SGLT2 inhibitor, has shown antioxidative, antiapoptotic, and anti-inflammatory effects in various experimental models [18]. While primarily known for its antihyperglycemic effects, recent studies reveal its broad impacts, including weight loss, cardiovascular benefits, and improvements in metabolic parameters [19,20]. Dapagliflozin’s antioxidant effects, such as reduced ROS production and modulation of Ca^2+^ influx, along with its anti-inflammatory properties, suggest its potential in mitigating CCl_4_-induced hepatotoxicity [21]. Human hepatocellular carcinoma cells (HepG2) are known to express both SGLT-1 and SGLT-2 co-transporters [22]. Additionally, SGLT-2 has been observed in immortalized normal human hepatocyte-derived liver cells (L02) and immortalized human primary hepatocyte cells (HuS-E/2) [23]. Some of the in vitro studies have reported that SGLT-2 inhibitors exhibit anti-proliferative effects in various hepatocellular cell lines, partly by reducing glucose uptake [24,25]. Our study aimed to evaluate the hepatoprotective potential of dapagliflozin in an established model of CCl₄-induced liver injury and explore the molecular mechanisms of dapagliflozin’s effects by concentrating on the Nrf2/HO-1 signaling pathway. This research endeavor seeks to enhance comprehension and treatment strategies for oxidative stress-associated liver diseases.

## 2. Materials and Methods

### 2.1. Drugs and Reagents

The active pharmaceutical ingredient of silymarin was sourced from Sigma-Aldrich-Merck Limited, Bangalore, India. Dapagliflozin was acquired from AstraZeneca. Colorimetric assay kits for ALT, AST, and total bilirubin were obtained from Alliance Global, Dubai, United Arab Emirates. Fasting blood glucose glucometer strips were purchased from Life Pharmacy, Dubai, United Arab Emirates. Colorimetric assay kits for glutathione peroxidase, catalase, SOD, nitrites, and rat ELISA kits for 4-HNE, IL-1β, IL-6, TNF-α, Nrf2, and HO-1 were procured from Elabscience, United States through the Scientechnic, a distributor based in the UAE. All laboratory-grade chemicals including carbon tetrachloride were obtained through local distributors in the UAE.

### 2.2. Animals

Thirty adult female Wistar rats ranging from 8–10 weeks old and weighing 150–200 g were bred at the Central Animal Research Facility, Ras Al Khaimah Medical and Health Sciences University (RAKMHSU), UAE. These animals were housed in controlled conditions including a 12 h dark/12 h light cycle, temperatures between 22–24 °C, and relative air humidity of 40–60%. They had a regular supply of tap water and a normal rat pellet diet consisting of standard calories. After a week of acclimatization to the research animal holding room, the rats were randomly allocated into various groups. Ethics approval was taken from the RAKMHSU Research and Ethics Committee (RAKMHSU-REC-014-2022/23-UG-M).

### 2.3. Rationale for Dose Selection of Carbon Tetrachloride, Silymarin, and Dapagliflozin and Their Dissolution

Hepatotoxicity was induced by administering 1:1 mixture of CCl_4_ and olive oil; (1.59 mg/kg and 0.92 mg/kg, respectively, ~1 mL/kg; i.p. every 48 h) [11,12,14]. We have earlier reported the hepatoprotective dose of silymarin as 50 mg/kg/day for Wistar rats [7,9,11,12,13,14]. The US FDA-approved dose for the antidiabetic effect of dapagliflozin in humans is 10 mg/day. According to Paget and Barnes’ body surface area ratio, the human dose was converted to the rat dose equivalent of 0.9 mg/kg/day. Silymarin and dapagliflozin were each dissolved in 2% gum acacia and administered orally.

### 2.4. Experimental Design

Following the measurement of baseline body weight, 30 adult female Wistar rats (8–10 weeks old) were randomly divided into five groups (*n* = 6/group). The treatment regimen followed for 14 days between 10 and 11 AM every day is mentioned below:

Group I (Normal healthy control): Olive oil (1 mL/kg; i.p. every 48 h) + 2% gum acacia (1 mL/kg/day; p.o.)

Group II (Negative control): CCl_4_-intoxicated hepatotoxic control rats (1:1 mixture of CCl_4_ and olive oil; 1 mL/kg; i.p. every 48 h) + 2% gum acacia (1 mL/kg/day; p.o.)

Group III (Positive control; CCl_4_ + silymarin): CCl_4_-intoxicated hepatotoxic rats (1:1 mixture of CCl_4_ and olive oil; 1 mL/kg; i.p. every 48 h) + silymarin (50 mg/kg/day; p.o.)

Group IV (Test; CCl_4_ + dapagliflozin): CCl_4_-intoxicated hepatotoxic rats (1:1 mixture of CCl_4_ and olive oil; 1 mL/kg; i.p. every 48 h) + dapagliflozin (0.9 mg/kg/day; p.o.)

Group V (Test; CCl_4_ + silymarin + dapagliflozin): CCl_4_-intoxicated hepatotoxic rats (1:1 mixture of CCl_4_ and olive oil; 1 mL/kg; i.p. every 48 h) + silymarin (50 mg/kg/day; p.o. + dapagliflozin 0.9 mg/kg/day; p.o.)

The body weight was monitored weekly during the experiment. On 15th day, overnight fasted experimental rats were anesthetized by administering ketamine (60 mg/kg) and xylazine (10 mg/kg) intraperitoneally. Fasting blood glucose was estimated by glucose oxidase–peroxide reactive strips using a glucometer after the fasting blood samples were obtained from their tail vein (tail tip).

### 2.5. Collection of Blood and Serum Preparation

Blood was collected from the retro-orbital plexus of veins using capillary tubes and transferred into microcentrifuge tubes. The serum was then isolated from the whole blood by centrifugation at 3000 rpm at 4 degrees Celsius (°C) for 20 min in a cooling centrifuge. Subsequently, the supernatant was stored at −80 degrees Celsius (°C) for biochemical analysis.

### 2.6. Collection of the Liver and Its Gross Examination

Anesthetized animals were euthanized after the blood collection. Animals were placed in a recumbent supine position on the animal operation table. An incision was made on the ventral aspect of the anterior abdominal wall by using a surgical scalpel to open the abdominal cavity. The liver was collected from the right upper quadrant of the abdomen by dissecting it from the abdominal muscles, fascia, visceral fats, and major blood vessels. Gross morphological examination of the liver was performed. The liver was then washed in regular saline and soaked on blotting paper to extract the blood. Half of the liver was used to prepare its homogenate for biochemical estimations, and the other half was kept in 10% formalin for histopathological analysis.

### 2.7. Liver Homogenate Preparation

A 10% liver homogenate was prepared in a cold potassium phosphate buffer of 50 mM concentration and pH 7.4 using tissue homogenizer. Following further centrifugation at 3000 rpm for 10 min, the resultant supernatant was stored at −80 degrees Celsius (°C).

### 2.8. Biochemical Estimations in Serum and Liver Homogenates

ALT, AST, and total bilirubin levels in the serum were determined using standard protocols outlined in their respective assay kits, employing a colorimetric method with an autoanalyzer. SOD, GSH-Px, CAT, and nitrite levels were measured following colorimetric assay protocols provided with the kits, with optical density readings taken at 540 nm, 340 nm, 405 nm, and 550 nm, respectively, using a microplate reader.

Rat-specific 4-HNE levels in liver homogenate were estimated using Competitive ELISA principle. The micro ELISA plate provided was pre-coated with 4-HNE. During the reaction, 4-HNE in samples or standards competed with a fixed amount of 4-HNE on the solid phase supporter for sites on the Biotinylated Detection Ab specific to 4-HNE. Excess conjugate and unbound samples or standards were washed, followed by the addition of avidin conjugated to horseradish peroxidase (HRP) to each well and incubation. Subsequently, a TMB substrate solution was added to each well. The enzyme–substrate reaction was halted with stop solution, and the color change was measured spectrophotometrically at a wavelength of 450 nm using a microplate reader. 4-HNE concentration in the samples was determined by comparing their OD to the standard curve.

Rat-specific Nrf2, HO-1, IL-1β, IL-6, and TNF-α levels in liver homogenate were assessed via sandwich ELISA principle. The provided micro ELISA plates were pre-coated with antibodies specific to rat Nrf2/HO-1/IL-1β/IL-6/TNF-α. Samples (or standards) were added to the wells and combined with the specific antibody. Subsequently, a biotinylated detection antibody specific for rat Nrf2/HO-1/IL-1β/IL-6/TNF-α and avidin horseradish peroxidase (HRP) conjugate was added sequentially to each well and incubated. After washing away free components, substrate solution was added to each well. Wells containing rat Nrf2/HO-1/IL-1β/IL-6/TNF-α, biotinylated detection antibody, and avidin-HRP conjugate exhibited a blue coloration. The enzyme–substrate reaction was stopped with stop solution, resulting in a yellow color change. Optical density (OD) was measured spectrophotometrically at 450 nm using a microplate reader, with OD values proportional to the concentration of rat Nrf2/HO-1/IL-1β/IL-6/TNF-α. Concentrations in the samples were calculated by comparing their OD to the standard curve.

### 2.9. Qualitative Histopathological Examination of Liver

Liver tissue specimens were obtained from every group and fixed in a 10% phosphate-buffered formalin solution. Furthermore, a small portion of each liver sample was cut and dehydrated using increasing concentrations of ethyl alcohol (50% for 24 h, 70% for 24 h, 90% for 12 h, and 100% for 12 h), cleared with 99.14% xylene until the tissues became transparent and embedded in molten paraffin wax. After 24 h, 6-micron-thick paraffin sections were sliced using a microtome and affixed onto albumenized glass slides, with appropriate labeling. These sections underwent de-waxing in 99.14% xylene for 10 min, followed by hydration through decreasing ethyl alcohol concentrations for 2 min in each 100%, 90%, 70%, and 50% and finally in the distilled water for 10 min. Subsequently, the sections were stained with commercially prepared Harris hematoxylin for 5 min. Thereafter, sections were kept in running tap water for 10 min. Further, staining was performed with 2% eosin for 2 min. Later, sections were washed in 90% alcohol for 2 min and in 100% alcohol for another 2 min. Later, sections were cleared in 99.14% xylene. Finally, 2–3 drops of DPX mountant were applied onto the slides, and coverslips were gently placed to prevent tissue drying. The prepared slides were then examined for any morphological changes under a light microscope (Olympus BX53, Olympus Life Science Solutions, Tokyo, Japan) at 100× and 400× magnifications. Later, photomicrographs of the liver cell slides were captured and qualitatively analyzed.

### 2.10. Statistical Analysis

Using SPSS version 29, normally distributed data were presented as mean ± standard deviation. Thereafter, two-way analysis of variance (ANOVA) was conducted followed by a post hoc Tukey’s test. Statistical significance was defined as *p* < 0.05.

## 3. Results

### 3.1. Impact on Liver Function Test

**Figure 1:**
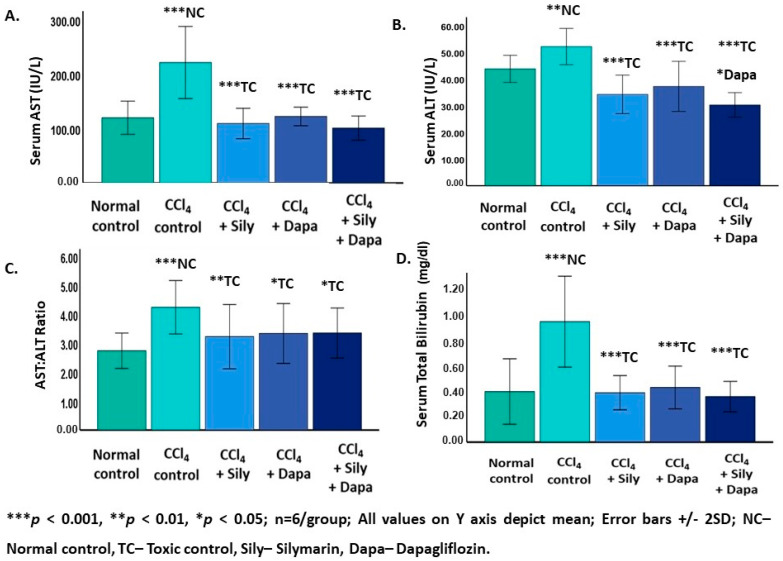
Effect on liver function test. (**A**) Effect on aspartate aminotransferase (AST); (**B**) Effect on alanine aminotransferase (ALT); (**C**) Effect on the ration of aspartate aminotransferase (AST) and alanine aminotransferase (ALT); (**D**) Effect on total bilirubin.

The liver function test revealed a significant increase in serum ALT (52.13 ± 3.39; *p* = 0.002), AST (221.41 ± 33.13; *p* < 0.001), AST:ALT ratio (4.23 ± 0.46; *p* < 0.001), and TB levels (0.87 ± 0.16; *p* < 0.001) for the CCl_4_-intoxicated hepatotoxic control group in contrast to the normal control rats (ALT: 43.78 ± 2.53; AST: 119.86 ± 15.24; AST:ALT ratio: 2.73 ± 0.30; and TB: 0.36 ± 0.11). On the other hand, the administration of dapagliflozin to the CCl_4_-intoxicated hepatotoxic rats led to a considerable decrease in ALT (37.22 ± 4.64; *p* < 0.001), AST (122.29 ± 8.51; *p* < 0.001), AST:ALT ratio (3.33 ± 0.51; *p* = 0.018), and TB (0.39 ± 0.07; *p* < 0.001) in comparison to the CCl_4_-intoxicated hepatotoxic control group. Silymarin demonstrated a marked reduction in ALT (34.22 ± 3.61; *p* < 0.001), AST (109.26 ± 13.97; *p* < 0.001), AST:ALT ratio (3.22 ± 0.55; *p* = 0.007), and TB (0.36 ± 0.06; *p* < 0.001) compared to the CCl_4_-intoxicated hepatotoxic control rats. Interestingly, combined treatment with dapagliflozin and silymarin resulted in a notable decrease in serum levels of ALT (30.27 ± 2.29; *p* < 0.001), AST (100.85 ± 11.09; *p* < 0.001), AST:ALT ratio (3.34 ± 0.43; *p* = 0.021), and TB (0.33 ± 0.05; *p* < 0.001) compared to the CCl_4-_-intoxicated hepatotoxic control group. Furthermore, the CCl_4_-intoxicated hepatotoxic rats treated with a combination of both silymarin and dapagliflozin exhibited a notable decrease in ALT levels (*p* = 0.013) compared to the dapagliflozin alone-treated hepatotoxic rats (Figure 1).

### 3.2. Effect on Fasting Blood Glucose Levels, Body Weight, and Mortality Rate

During the experimental period, no significant changes (*p* > 0.05) were observed in either fasting blood glucose levels or body weight among all the experimental animals. Additionally, there were no instances of mortality recorded during the experiment.

### 3.3. Influence on Inflammatory Cytokines

**Figure 2:**
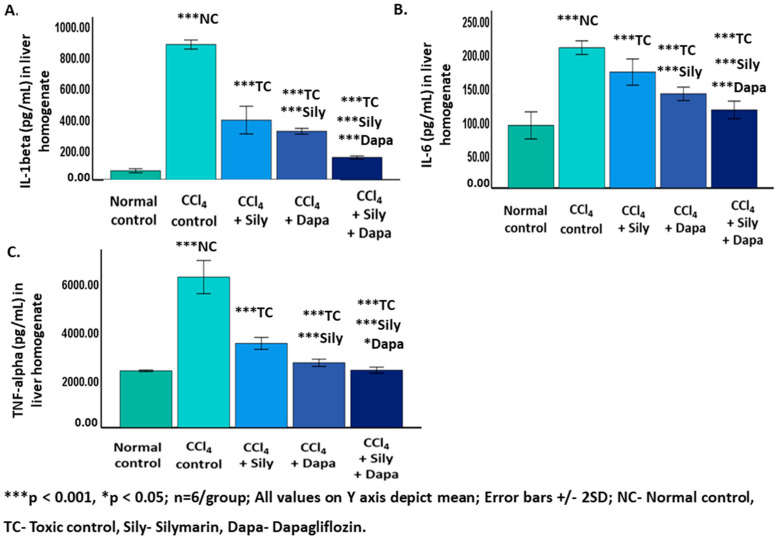
Effect on inflammatory cytokines. (**A**) Effect on IL-1beta; (**B**) Effect on IL-6; (**C**) Effect on TNF-alpha.

A significant increase (*p* < 0.001) in IL-1 beta (874.43 ± 14.64), IL-6 (208.19 ± 5.08), and TNF-alpha (6223.86+343.45) was noted in the CCl_4_-intoxicated hepatotoxic control rats in contrast to the normal control (IL-1 beta: 59.21 ± 6.61 and IL-6: 92.91 ± 10.04; 2352.74 ± 17.53). Furthermore, the administration of dapagliflozin to the CCl_4_-intoxicated hepatotoxic rats resulted in a notable reduction (*p* < 0.001) in IL-1 beta (314.37 ± 9.38), IL-6 (139.77 ± 4.97), and TNF-alpha (2684.21 ± 74.83) in contrast to both the CCl_4_-intoxicated hepatotoxic control and silymarin-treated hepatotoxic groups. Silymarin exhibited a significant decrease (*p* < 0.001) in IL-1 beta (386.39 ± 44.79), IL-6 (172.08 ± 9.73), and TNF-alpha (3480.29 ± 124.17) compared to the CCl_4_-intoxicated hepatotoxic control group. Notably, a combined treatment with dapagliflozin and silymarin in the CCl_4_-intoxicated hepatotoxic group demonstrated a significant decrease (*p* < 0.001) in IL-1 beta (145.05 ± 5.07), IL-6 (115.95 ± 6.50), and TNF-alpha (2376.81 ± 64.04) in contrast to the CCl_4_-intoxicated hepatotoxic control rats. Furthermore, the CCl_4_-intoxicated hepatotoxic group treated with a combination of silymarin and dapagliflozin displayed a significant decline (*p* < 0.001) in IL-1 beta, IL-6, and TNF-alpha in contrast to the silymarin alone-treated and dapagliflozin alone-treated hepatotoxic groups (Figure 2).

### 3.4. Effect on Oxidative Stress Biomarkers

**Figure 3:**
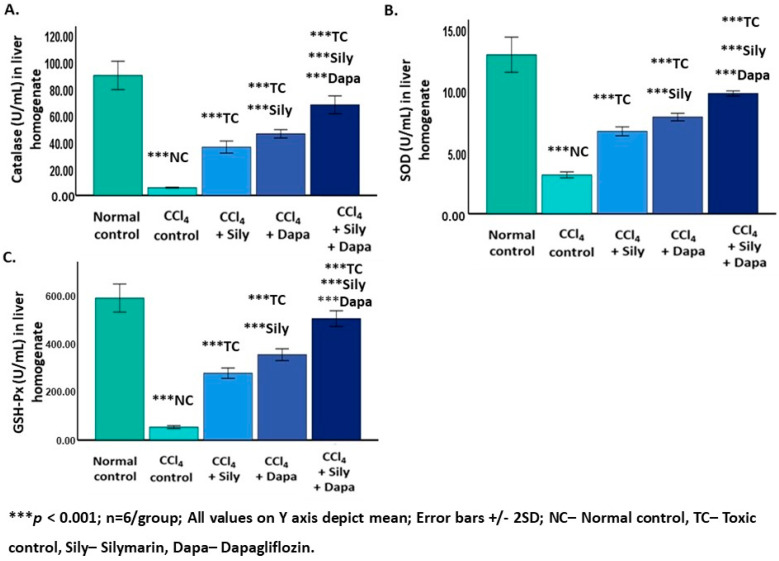
Effect on enzymatic antioxidants. (**A**) Effect on catalase; (**B**) Effect on superoxide dismutase; (**C**) Effect on glutahione peroxidase.

**Figure 4:**
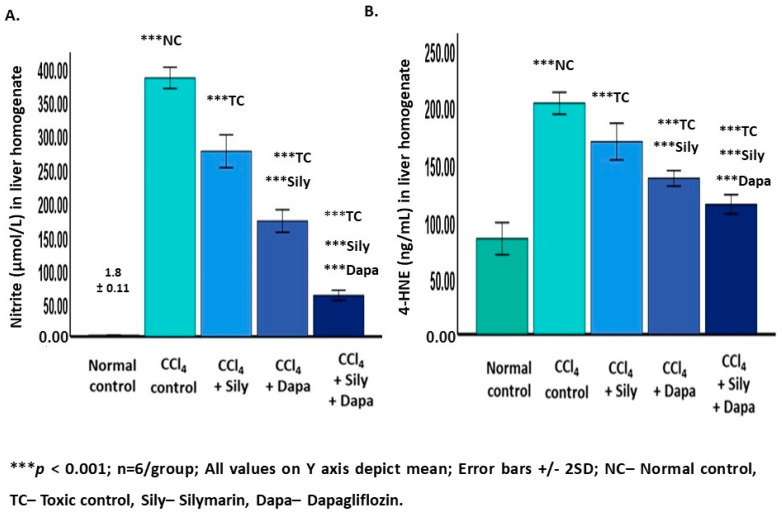
Effect on non-enzymatic antioxidants. (**A**) Effect on nitrite; (**B**) Effect on 4-HNE.

A significant increase (*p* < 0.001) in nitrites (383.03 ± 7.88) and 4-HNE (201.86 ± 4.79), along with a reduction (*p* < 0.001) in CAT (5.79 ± 0.16) and SOD (3.15 ± 0.12), and GSH-Px (2.95 ± 1.20), was observed in the CCl_4_-intoxicated hepatotoxic control rats compared to the normal control group (nitrites: 1.88 ± 0.11; 4-HNE: 83.58 ± 7.01; CAT: 89.55 ± 5.28; SOD: 12.92 ± 0.71; and GSH-Px: 29.03 ± 11.85). Conversely, administering dapagliflozin to these CCl_4_-intoxicated hepatotoxic rats resulted in a notable decrease (*p* < 0.001) in nitrites (171.08 ± 8.38) and 4-HNE (136.27 ± 3.39), and an increase (*p* < 0.001) in CAT (46 ± 1.54), SOD (7.86 ± 0.15), and GSH-Px (11.98 ± 4.89) compared to both the CCl_4_-intoxicated hepatotoxic control and silymarin-treated hepatotoxic groups. Silymarin exhibited a significant reduction (*p* < 0.001) in nitrites (274 ± 12.16) and 4-HNE (168.41 ± 8.03) and an elevation (*p* < 0.001) in CAT (36.08 ± 2.25), SOD (6.69 ± 0.18), and GSH-Px (10.76 ± 4.39) compared to the CCl_4_-intoxicated hepatotoxic control group. Notably, the combined treatment with dapagliflozin and silymarin in the CCl_4_-intoxicated hepatotoxic group showed a significant decrease (*p* < 0.001) in nitrites (61.06 ± 3.74) and 4-HNE (113.61 ± 4.18) and an increase (*p* < 0.001) in CAT (67.60 ± 3.33), SOD (9.77 ± 0.10), and GSH-Px (16.25 ± 6.63) in contrast to the CCl_4_-intoxicated hepatotoxic control rats. Additionally, the CCl_4_-intoxicated hepatotoxic group treated with a combination of silymarin and dapagliflozin exhibited a significant decrease (*p* < 0.001) in nitrites and 4-HNE and an increase (*p* < 0.001) in CAT, SOD, and GSH-Px in contrast to the silymarin alone-treated and dapagliflozin alone-treated hepatotoxic groups (Figure 3 and Figure 4).

### 3.5. Modulation of Nrf2/HO-1 Signaling Pathway

**Figure 5:**
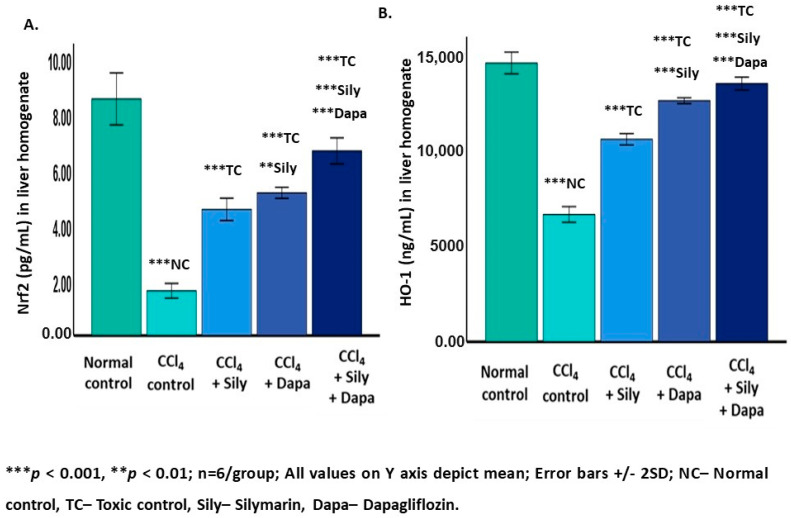
Effect on Nrf2 and HO-1. (**A**) Effect on Nrf2; (**B**) Effect on HO-1.

The modulation of the Nrf2/HO-1 signaling axis showed notable changes in response to various treatments. In CCl_4_-intoxicated hepatotoxic control rats, there was a significant decline (*p* < 0.001) in Nrf2 (6589.59 ± 203.2) and HO-1 (1.61 ± 0.13) levels in contrast to the normal control group (Nrf2: 14,426 ± 280.78 and HO-1: 8.52 ± 0.46). On the other hand, treatment of the CCl_4_-intoxicated hepatotoxic rats with dapagliflozin showed a remarkable increase (*p* < 0.001) in Nrf2 (12,474.40 ± 76.06) and HO-1 (5.14 ± 0.09) levels in contrast to the CCl_4_-intoxicated hepatotoxic control groups. Nrf2 (*p* < 0.001) and HO-1 (*p* = 0.005) were significantly increased in the hepatotoxic rats treated with dapagliflozin compared to the silymarin-treated hepatotoxic group. Silymarin alone exhibited a significant elevation (*p* < 0.001) in Nrf2 (10,476.30 ± 146.5) and HO-1 (4.54 ± 0.20) levels compared to the CCl_4_-intoxicated hepatotoxic control rats. Interestingly, the combination of silymarin and dapagliflozin significantly elevated (*p* < 0.001) the levels of both Nrf2 (13,360.01 ± 161.23) and HO-1 (6.65 ± 0.23) in contrast to the CCl_4_-intoxicated hepatotoxic control and the silymarin alone-treated and dapagliflozin alone-treated hepatotoxic groups (Figure 5).

### 3.6. Impact on Gross Examination of the Liver

**Figure 6:**
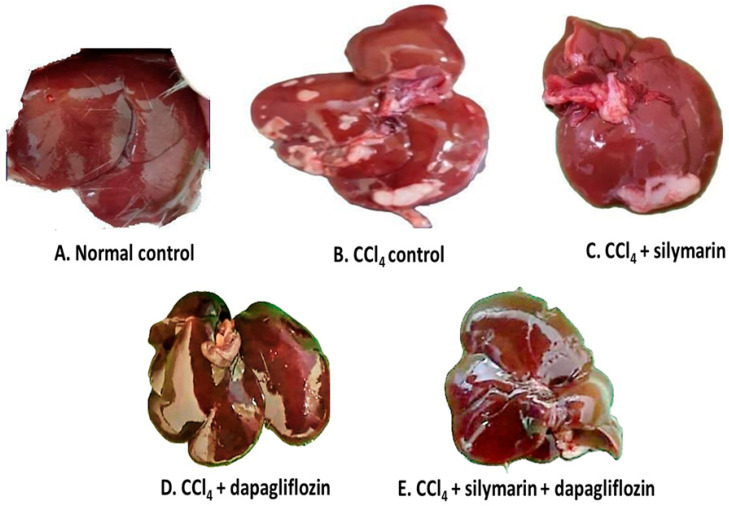
Gross examination of liver.

Upon gross examination of the liver, we observed that the normal healthy control group exhibited a dark reddish-brown liver with a soft, smooth, and shiny surface, whereas, CCl_4_-induced hepatotoxic control rats showed a slight pale color of the liver along with multiple yellowish-white patches indicating fatty infiltration over the surface. Silymarin alone-treated hepatotoxic rats displayed comparatively fewer yellowish-white patches than the hepatotoxic control group. Surprisingly, hepatotoxic rats treated with dapagliflozin alone, as well as in combination with silymarin, demonstrated normal liver morphology, similar to that of the normal control group (Figure 6).

### 3.7. Effect on Cellular Architecture of Liver

**Figure 7:**
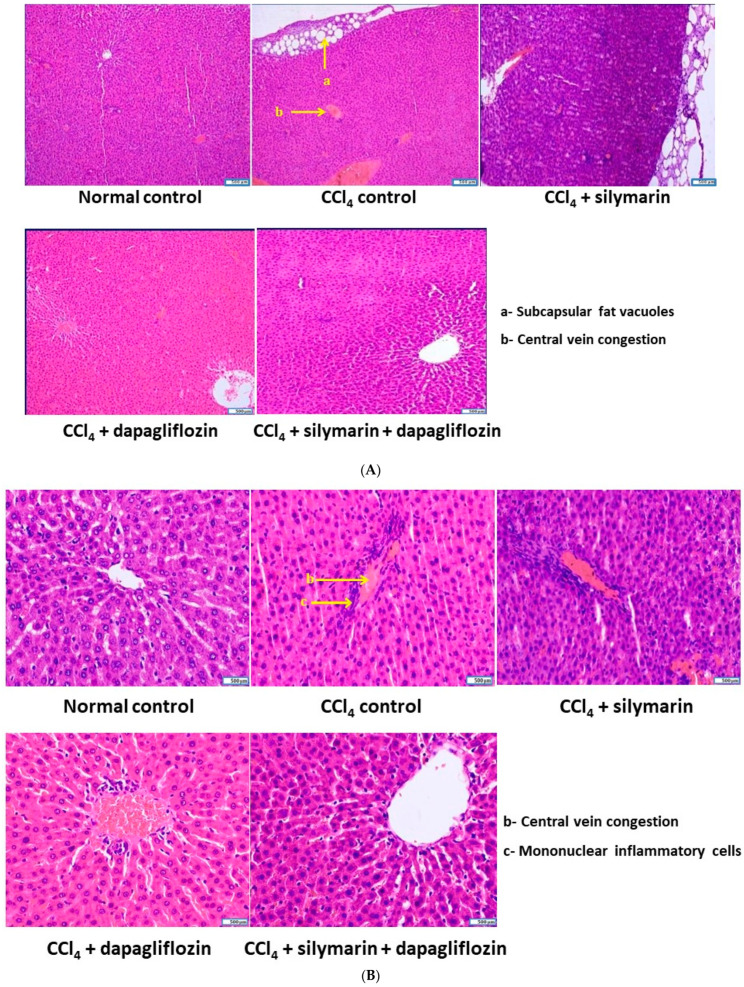
(**A**) Qualitative histopathological examination of liver (stained with H & E and observed under 100× magnification). (**B**) Qualitative histopathological examination of liver (stained with H & E and observed under 400× magnification).

The normal healthy control group displayed a typical hepatocellular structure, with hepatocytes arranged in hepatic cords concentrically around the central vein. In contrast, the CCl_4_-induced hepatotoxic control group exhibited subcapsular fat vacuoles, a dilated and congested central vein, and moderate perivenular and periportal infiltration of mononuclear cells, mainly macrophages and lymphocytes. Silymarin alone-treated hepatotoxic rats showed mildly dilated central veins, mild to moderate perivenular mononuclear cell infiltration, and subcapsular fat vacuoles. Dapagliflozin alone-treated hepatotoxic rats demonstrated infiltration of a few mononuclear inflammatory cells around the central vein, along with significant restoration of hepatocyte architecture. Hepatotoxic rats treated with a combination of dapagliflozin and silymarin had a hepatocellular architecture closely resembling that of the normal control group (Figure 7A,B).

## 4. Discussion

This study employed a comprehensive approach, including biochemical, histological, and molecular analyses, to uncover the molecular mechanisms that protect against carbon tetrachloride-induced hepatotoxicity through the use of dapagliflozin. The results enhance our understanding of dapagliflozin’s varied effects and provide detailed insights into the hepatoprotective properties of both dapagliflozin and silymarin in the context of CCl_4_-induced liver damage. This is demonstrated by changes in liver function, inflammatory markers, oxidative stress biomarkers, modulation of the Nrf2/HO-1 signaling pathway, and noticeable gross and histopathological changes in the liver.

Carbon tetrachloride (CCl_4_) has been widely used in experimental models to investigate the cellular mechanisms behind oxidative damage [26]. CCl_4_ is activated by cytochrome P-4502E, 2B1, 2B2, and possibly CYP 3A to form the trichloromethyl radical (CCl3^•^) and trichloromethyl peroxy radical (CCl3OO^•^), leading to lipid peroxidation and subsequent tissue damage [8]. Enhanced lipid peroxidation, coupled with the depletion of antioxidants in tissues, results in structural changes in the endoplasmic reticulum and other membranes, loss of metabolic enzyme activation, reduced protein synthesis, and elevated levels of serum transaminases, total bilirubin, and conjugated bilirubin, culminating in liver damage [27]. The leakage of cellular enzymes into plasma indicates hepatic tissue damage. Alanine transaminase (ALT) is considered an important diagnostic marker of liver injury [28]. The marked increase in serum ALT, AST, AST:ALT ratio, and total bilirubin in the CCl_4_-intoxicated hepatotoxic control rats highlights the extent of liver damage. Dapagliflozin, both alone and in combination with silymarin, exhibited a significant decrease in these indicators, suggesting a protective effect on the liver. Our observations concerning dapagliflozin’s positive impacts on liver function align with studies emphasizing its ability to mitigate non-alcoholic fatty liver disease in diabetic animal models [29,30,31]. These findings are consistent with our previous reports on CCl_4_-induced elevation of hepatic injury biomarkers in serum [11,12,14].

Notably, the combined treatment of dapagliflozin and silymarin significantly enhanced liver function, suggesting a synergistic effect. Gross and histopathological examinations of liver tissues further support the hepatoprotective effects of dapagliflozin and silymarin. Rats treated with either dapagliflozin or silymarin, especially in the combination group, exhibited a restoration of liver structure resembling the healthy control group. This observation is consistent with biochemical markers, reinforcing the potential therapeutic benefits of these interventions.

CCl_4_-induced hepatotoxicity is characterized by elevated pro-inflammatory cytokines [32]. Oxidative stress can elevate levels of inflammatory cytokines. Reactive oxygen species (ROS) generated during oxidative stress can activate nuclear factor kappa B (NF-κB), a key transcription factor that promotes the expression of various pro-inflammatory cytokines such as tumor necrosis factor-alpha (TNF-α), interleukin-1 beta (IL-1β), and interleukin-6 (IL-6). This link between oxidative stress and inflammation underscores the intricate interplay between oxidative damage and the immune response, contributing to the pathogenesis of various diseases [33]. Dapagliflozin exhibited a significant reduction in IL-1 beta, IL-6, and TNF-alpha, aligning with studies emphasizing its anti-inflammatory effects [34,35,36]. The combination of dapagliflozin and silymarin displayed a robust suppression of inflammatory biomarkers, indicating a potential synergistic effect.

Oxidative stress is pivotal in CCl_4_-induced liver damage [7,8]. Dapagliflozin significantly reduced nitrites and 4-hydroxynonenal (4-HNE), while increasing antioxidant enzymes such as superoxide dismutase (SOD), catalase (CAT), and glutathione peroxidase (GSH-Px), supporting studies that highlight dapagliflozin’s antioxidative potential [37,38] and silymarin’s effects [39]. The combination therapy showed a comprehensive improvement, underscoring a synergistic effect in combating oxidative stress. Nrf2, a protective transcription factor sensitive to redox changes, regulates detoxification gene activation, safeguarding cells from oxidative stress. During oxidative stress, Nrf2 dissociates from Keap1 and enters the nucleus, where it binds to antioxidant response elements (AREs) in the promoter region of genes encoding numerous antioxidant enzymes like SOD, GSH-Px, CAT, and phase II detoxification enzymes, such as NAD(P)H quinone dehydrogenase 1 (NQO1), to counteract oxidative stress [40]. The Nrf2/HO-1 pathway coordinates the antioxidant response, playing critical roles in cellular defense against oxidative stress and inflammation. Activation of this pathway upregulates various antioxidant and cytoprotective genes, contributing to cellular homeostasis. Nrf2 activation has been shown to enhance the expression of SOD isoforms, including SOD1, SOD2, and SOD3, in response to oxidative stress [41]. These enzymes scavenge superoxide radicals and are crucial in cellular antioxidant defense. Although direct evidence linking Nrf2 to catalase regulation is limited, studies show the interplay between Nrf2 and catalase in protecting cells from oxidative stress [42]. Nrf2 activation indirectly enhances catalase activity by modulating the cellular redox environment. Activation of Nrf2 also leads to the transcriptional upregulation of GSH-Px, as demonstrated in various studies [43]. GSH-Px enzymes reduce hydrogen peroxide and organic hydroperoxides using reduced glutathione, thus protecting cells from oxidative damage. The Nrf2/HO-1 pathway has been shown to reduce nitrite levels indirectly by decreasing oxidative stress and inflammation [44]. Activation of Nrf2 inhibits nitric oxide (NO) production and oxidative stress-induced nitrosative damage. Activation of the Nrf2/HO-1 pathway mitigates lipid peroxidation and reduces 4-HNE accumulation in various cellular models [45]. Nrf2 activation induces the expression of HO-1, which inhibits 4-HNE-induced oxidative damage by promoting its metabolism [46,47,48].

In our study, CCl_4_ exposure resulted in a notable decrease in Nrf2 and HO-1 levels, indicating compromised antioxidant defense. Dapagliflozin significantly modulated these markers, highlighting its role in enhancing the Nrf2/HO-1 pathway. The combination therapy exhibited strong activation of the Nrf2/HO-1 axis, suggesting a potential mechanism for the observed antioxidative effects, including increased levels of enzymatic antioxidant parameters such as GSH-Px, SOD, and CAT along with decreased levels of non-enzymatic antioxidant parameters like 4-HNE and nitrites. The amelioration of a CCl_4_-induced decline in liver function, inflammation, oxidative stress, and normalization of hepatocellular architecture may be attributed to the upregulation of Nrf2/HO-1 by dapagliflozin alone and in combination with silymarin. Our results underscore the therapeutic promise of focusing on Nrf2/HO-1 in addressing different pathological conditions linked to oxidative stress and inflammation. One of the studies reported that dapagliflozin recovered cholesterol metabolism functions in type 2 diabetes mellitus (T2DM) mice liver via activating the antioxidant Nrf2/HO-1 pathway, highlighting the involvement of this pathway in dapagliflozin-mediated hepatoprotection protection [49]. Emerging evidence suggests that dapagliflozin exerts its hepatoprotective effects through modulation of key signaling pathways. The Nrf2/HO-1 pathway, known for its role in cellular defense against oxidative stress, appears to be activated by dapagliflozin.

Our study boasts several strengths, employing a multidimensional approach encompassing biochemical, histological, and molecular analyses to thoroughly evaluate liver function, inflammation, oxidative stress, and underlying molecular mechanisms. We investigated the potential repurposing effect of the well-established antidiabetic drug dapagliflozin at a dosage of 0.9 mg/kg/day, equivalent to the human therapeutic dose of 10 mg/day, without compromising safety considerations. Our research delves into the mechanistic underpinnings of the hepatoprotective effects, particularly emphasizing the modulation of the nuclear erythroid 2-related factor 2/heme oxygenase-1 signaling pathway, which enhances antioxidant and anti-inflammatory responses. However, our study does have limitations. These include a small sample size and the use of a rat model of CCl_4_-induced hepatotoxicity potentially limiting its representation of human physiology and pathophysiology and thus restricting the generalizability of the findings to clinical settings. Although our study sheds light on the hepatoprotective potential of dapagliflozin and its combination with silymarin in carbon tetrachloride-induced hepatotoxicity, addressing the aforementioned limitations would bolster the robustness and applicability of our findings.

## 5. Conclusions

The present study reveals the substantial hepatoprotective potential of dapagliflozin alone and in combination with silymarin in carbon tetrachloride-induced hepatotoxicity model in Wistar rats by upregulating the Nrf2/HO-1 signaling pathway. Silymarin and dapagliflozin combination therapy demonstrated synergistic effects across various parameters, surpassing the individual effects of dapagliflozin and silymarin. These promising findings suggest that co-administering dapagliflozin and silymarin could be an effective therapeutic strategy for mitigating CCl4-induced hepatotoxicity. This study is significant as it explores a novel therapeutic use for dapagliflozin beyond its established role in diabetes management. Exploring the translational potential of dapagliflozin and silymarin in combating hepatotoxicity holds promise for improving clinical outcomes.

## Data Availability

All data arising from this study are included within the article.

## Abbreviations

CCl_4_—Carbon tetrachloride; ROS—Reactive oxygen species; CAT—Catalase; GSH-Px—Glutathione peroxidase; SOD—Superoxide dismutase; Nrf2—Nuclear factor erythroid 2-related factor 2; HO-1—Heme oxygenase 1; ARE—Antioxidant response element, TNF-α—Tumor necrosis factor alpha; IL-1β—Interleukin 1 beta; IL-6—Interleukin-6; SGLT2—Sodium–glucose transport protein 2; 4-HNE—4-Hydroxynonenal; p.o.—Per oral; i.p.—Intraperitoneal; H & E—Hematoxylin and Eosin; DPX—Dibutylphthalate polystyrene xylene; Keap1—Kelch-like ECH-associated protein 1; NQO1—NAD(P)H quinone dehydrogenase 1; TMB- 3,3′,5,5′—Tetramethylbenzidine; SPSS—Statistical package for social sciences; RAKMHSU—RAK Medical and Health Sciences University; REC—Research and Ethics Committee; US—United States; UAE—United Arab Emirates; FDA—Food and Drug Administration.
